# Optimized ROI size on ADC measurements of normal pancreas, pancreatic cancer and mass-forming chronic pancreatitis

**DOI:** 10.18632/oncotarget.18457

**Published:** 2017-06-12

**Authors:** Chao Ma, Jing Li, Mbaiaoure Barak Boukar, Panpan Yang, Li Wang, Luguang Chen, Li Su, Jianxun Qu, Shi-Yue Chen, Qiang Hao, Jian-Ping Lu

**Affiliations:** ^1^ Department of Radiology, Changhai Hospital of Shanghai, The Second Military Medical University, Shanghai, China; ^2^ School of Pharmacy, Second Military Medical University, Shanghai, China; ^3^ GE Healthcare, MR Group, Shanghai, China

**Keywords:** DWI, apparent diffusion coefficient (ADC), pancreatic cancer, region of interest (ROI), chronic pancreatitis

## Abstract

**Objectives:**

To investigate the effects of region of interest (ROI) sizes on apparent diffusion coefficient (ADC) measurements for the differentiation of normal pancreas (NP), pancreatic ductal adenocarcinoma (PDAC) and mass-forming chronic pancreatitis (MFCP).

**Results:**

There were no significant differences for the mean ADCs measured by 12 different-size ROIs for MFCP, or PDAC and NP (*P* = 0.858–1.0). With the increase of ROI size (≥ 55 mm^2^), ADCs of PDAC were significantly lower than those of NP (all *P* < 0.05), but there was no difference of the accuracy in ADC for differentiating the two groups only at a ROI size of 214 mm^2^. When ROI size was above 99 mm^2^, ADCs of MFCP were significantly lower than those of NP (all *P* < 0.05). There were no significant differences for any of the mean ADCs measured by 12 different-size ROIs between PDAC and MFCP (*P* > 0.05).

**Materials and Methods:**

Diffusion-weighted imaging (DWI) was performed on 89 participants: 64 with PDAC, 7 with MFCP, as well as 18 healthy volunteers. ADC maps were created using mono-exponential model. A homemade software was used to measure the mean ADC values of 12 concentric round ROIs (areas: 15, 46, 55, 82, 99, 121, 134, 152, 161, 189, 214, 223, and 245 mm^2^) for the mass of lesions and the NP tissue.

**Conclusions:**

In ADC measurements, the optimized ROI size is 214 mm^2^ for the differentiation of PDAC and NP; ROI size of ≥ 99 mm^2^ is recommended to differentiate between MFCP and NP. ADC was not useful for the differentiation of PDAC and MFCP.

## INTRODUCTION

Differential diagnosis of mass-forming chronic pancreatitis (MFCP) and pancreatic ductal adenocarcinoma (PDAC) is of clinical significance due to the different treatment strategies [1, 2], and yet this remains challenging in practice because of the similarity of imaging presentations for the two different entities [3–6]. Diffusion-weighted imaging (DWI) with quantitative measurement of apparent diffusion coefficient (ADC) values provide an alternative to conventional anatomical magnetic resonance imaging (MRI), such as T1- (T1WI) and T2-weighted imaging (T2WI), for the detection and characterization of cystic and solid pancreatic tumors in clinical practice [7].

Some studies have been carried out to investigate the possibility to differentiate MFCP from PDAC by using qualitative DWI and quantitative ADC [7–12]. However, the use of mean ADC values to differentiate MFCP from PDAC may still be challenging, possibly due to the variable proportions of fibrosis and inflammation in MFCP, fibrosis and cell density in tumors, and literature data are inhomogeneous and controversial [8–12]. In terms of the measurement, this could be caused by the large variation in the region of interest (ROI) sizes [13, 14] in these studies (ranging from 19 to 879 mm^2^) [8–12]. The avoidance of the placement of smaller ROIs within lesions is commonly recommended, particularly for the response assessment studies [7, 15]. There is a clear need for the standardization of ROI sizes for ADC measurements of pancreatic diseases to enable the validation of this quantitative parameter as a qualified biomarker for longitudinal clinical trials. To our knowledge, the effect of ROI size on ADC measurements in normal pancreatic tissue or pancreatic lesions have rarely been studied. Thus, the aim of this study was to investigate the influences of ROI size in ADC measurements for the differentiation between normal pancreas (NP), PDAC and MFCP.

## RESULTS

**Figure 1:**
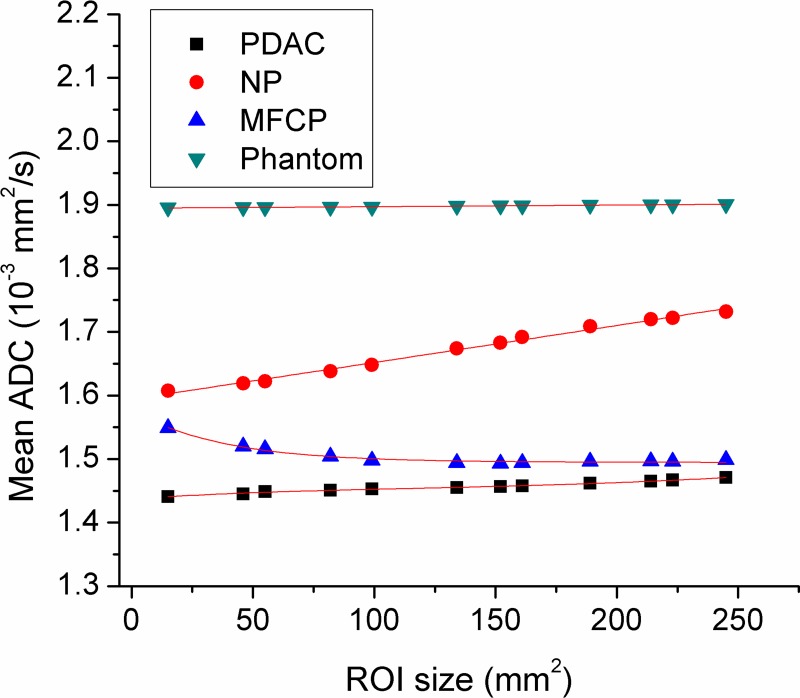
The mean apparent diffusion coefficient (ADC) curves for pancreatic ductal adenocarcinoma (PDAC), mass-formed chronic pancreatitis (MFCP), normal pancreas (NP) and water phantom

Two repeated DWI experiments of phantom using a standard eight-element phased array body coil for clinical examination revealed similar results. The mean ADCs of the water with different ROI sizes range from 1.895 ± 0.056 to 1.901 ± 0.053 × 10^−3^ mm^2^/s (Figure 1).

The mean ADC values of three participant groups (PDAC, MFCP and NP) with different ROI sizes are summarized and shown in Table 1. The typical averaged ADC curves of PDAC (64 cases), MFCP (7 cases), NP (18 cases) and water phantom (10 cases) with different ROI sizes are demonstrated in Figure 1.

**Table 1:** Mean ADC values (× 10^−^^3^ mm^2^/s) of PDAC, FP, NP measured by using 12 concentric round ROIs and comparisons of the mean values among 3 groups

ROI size (mm^2^)	PDAC (64)	MFCP (7)	NP(18)	*P*^#^
15	1.44 ± 0.28	1.55 ± 0.75	1.61 ± 0.30	0.103
46	1.45 ± 0.26	1.52 ± 0.72	1.62 ± 0.29	0.055
55	1.45 ± 0.29	1.52 ± 0.71	1.62 ± 0.28	0.048^*^
82	1.45 ± 0.25	1.50 ± 0.70	1.64± 0.26	0.039^*^
99	1.45 ± 0.25	1.50 ± 0.69	1.65 ± 0.25	0.026^**^
134	1.46 ± 0.24	1.49 ± 0.67	1.67 ± 0.24	0.011^**^
152	1.46 ± 0.24	1.49 ± 0.66	1.68± 0.23	0.006^**^
161	1.46 ± 0.24	1.49 ± 0.66	1.69± 0.24	0.005^**^
189	1.46 ± 0.24	1.50 ± 0.65	1.71± 0.23	0.003^**^
214	1.46 ± 0.24	1.50 ± 0.65	1.72± 0.23	0.001^**^
223	1.47 ± 0.23	1.50 ± 0.64	1.72± 0.22	0.001^**^
245	1.47 ± 0.23	1.50 ± 0.63	1.73± 0.22	0.001^**^

ADC, apparent diffusion coefficient; PDAC, pancreatic ductal adenocarcinoma; MFCP, mass-formed chronic pancreatitis; NP, normal pancreas; SD, standard deviation.

^*^Post-hoc analyses show significant differences between PDAC and NP (*P* < 0.05).

^**^Post-hoc analyses show significant differences both between PDAC and NP, FP and NP groups.

^#^Kruskal-Wallis tests.

ANOVA results revealed ROI size had no significant effects on the mean ADC values for all the three groups (*P* = 0.858–1.0). Comparisons of the mean ADC values were performed and the results demonstrated significant differences among the three participant groups while the ROI size was above 55 mm^2^. The multiple comparisons results demonstrated that the mean ADCs of PDAC were significantly lower than those of NP (all *P* < 0.05) at a ROI size of ≥ 55 mm^2^, in addition, the mean ADCs of MFCP were significantly lower than those of NP (all *P* < 0.05) while at a ROI size was ≥ 99 mm^2^. However, there were no significant differences between PDAC and MFCP for any of the mean ADCs measured by 12 different-size ROIs (all *P* > 0.05).

**Figure 2:**
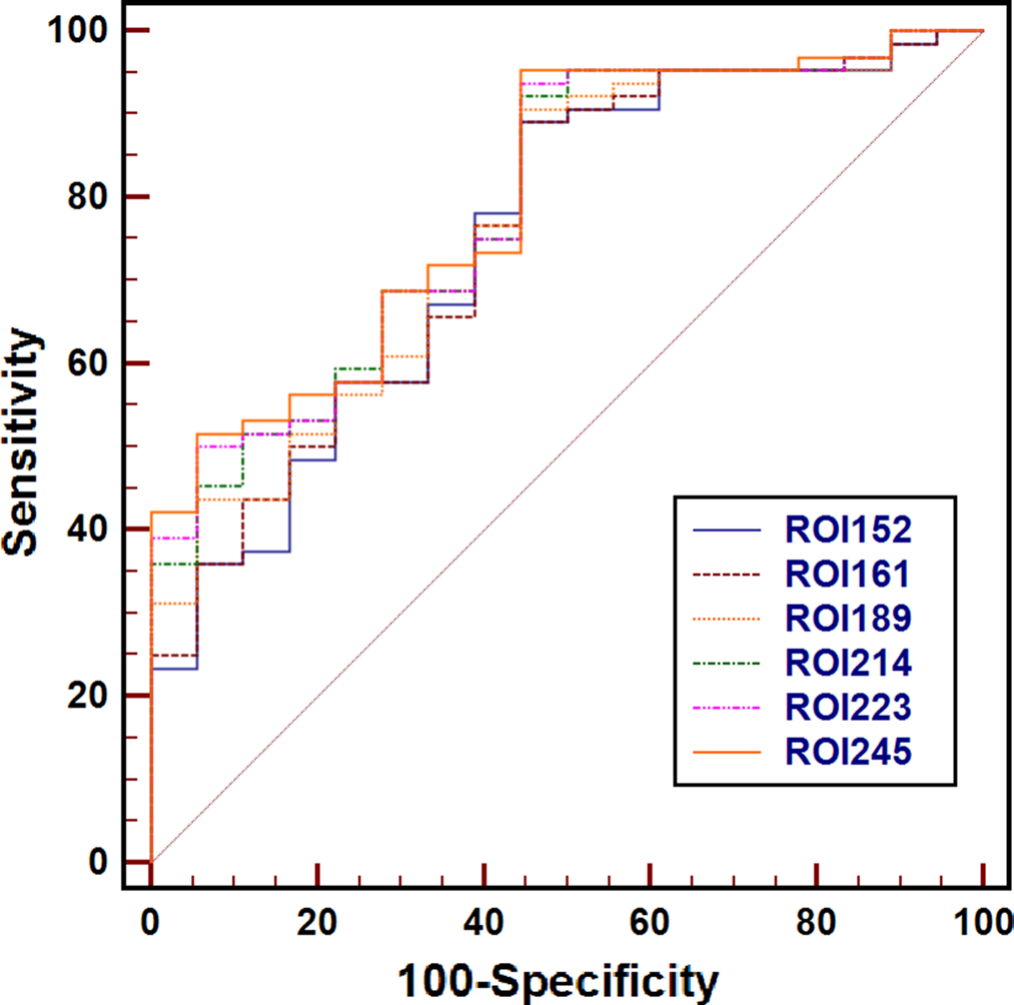
Receiver operating characteristic curves for the mean apparent diffusion coefficient measured with regions of interest (ROIs) of 152 mm^2^ to 245 mm^2^ for the differentiating between pancreatic ductal adenocarcinoma (PDAC) from normal pancreas (NP) There was no difference of the accuracy in ADC for differentiating between the PDAC and NP only at ROI size of 214 mm^2^ or higher.

ROC analyses results showed that there were no differences of the accuracy in ADC for differentiating between the PDAC and NP only at a ROI size of 214 mm^2^ (Table 2), and no difference of the accuracy in ADC were observed for differentiating between MFCP and NP while at a ROI size was ≥ 99 mm^2^ (Table 3, Figure 2).

**Table 2:** Results from the ROC analyses of the different ROIs derived mean ADCs to distinguish between pancreatic adenocarcinoma and healthy pancreas

ROI size (mm^2^)	Optimal cutoff values (× 10^−3^mm^2^/s)	AUC ± SE (95% CI)	Sensitivities (95% CI)	Specificities (95% CI)	PPV (%)	NPV (%)	ACC (%)
55	1.70	0.687 ± 0.079 (0.575−0.785)	89.1 (78.8 −95.5)	50.0 (26.0 −74.0)	86.4	56.3	80.5
82	1.72	0.694 ± 0.077 (0.582−0.791)	90.6 (80.7 −96.5)	50.0 (26.0 −74.0)	86.6	59.9	81.7
99	1.73	0.707± 0.074^*^ (0.596−0.802)	90.6 (80.7 – 96.5)	50.0 (26.0 −74.0)	86.6	59.9	81.7
134	1.74	0.731 ± 0.070 (0.622−0.823)	90.6 (80.7 – 96.5)	50.0 (26.0 −74.0)	86.6	59.9	81.7
152	1.70	0.751 ± 0.067 (0.643−0.840)	89.1 (78.8 – 95.5)	55.6 (30.8 −78.5)	87.7	58.9	81.7
161	1.70	0.756 ± 0.065 (0.649−0.844)	89.1 (78.8 – 95.5)	55.6 (30.8 −78.5)	87.7	58.9	81.7
189	1.75	0.770 ± 0.062 (0.664−0.856)	90.6 (80.7 – 96.5)	55.6 (30.8 −78.5)	87.9	62.5	82.9
214	1.79	0.788 ± 0.059 (0.684−0.871)	92.2 (82.7 – 97.4)	55.6 (30.8 −78.5)	88.1	66.7	84.2
223	1.79	0.793 ± 0.058 (0.689−0.874)	93.8 (84.8 – 98.3)	55.6 (30.8 −78.5)	88.3	71.6	85.4
245	1.79	0.800 ± 0.056 (0.697−0.881)	95.3 (86.9 – 99.0)	55.6 (30.8 −78.5)	88.4	76.9	86.6

ROC, Receiver operating characteristic curve; AUC, area under curve. ADC, apparent diffusion coefficient; SE, standard error; CI, confidence interval; ROI, region of interest; PPV, positive predictive value; NPV, negative predictive value; ACC, accuracy.

**Table 3:** Results from the ROC analyses of the different ROIs derived mean ADCs to distinguish between MFCP and healthy pancreas

ROI size (mm^2^)	Optimal cutoff values (× 10^−3^mm^2^/s	AUC ± SE (95% CI)	Sensitivities (95% CI)	Specificities (95% CI)	PPV (%)	NPV (%)	ACC (%)
99	1.51	0.659 ± 0.170^*^ (0.444−0.835)	71.4 (29.0 – 96.3)	72.2 (46.5 −90.3)	50.0	86.7	72.0
134	1.27	0.675 ± 0.174 (0.460−0.847)	57.1 (18.4 – 90.1)	100.0 (81.5 −100.0)	100	87.5	88.0
152	1.27	0.683 ± 0.170 (0.468−0.852)	57.1 (18.4 – 90.1)	100.0 (81.5 −100.0)	100	87.5	88.0
161	1.27	0.683 ± 0.170 (0.468−0.852)	57.1 (18.4 – 90.1)	100.0 (81.5 −100.0)	100	87.5	88.0
189	1.27	0.690 ± 0.170 (0.476−0.858)	57.1 (18.4 – 90.1)	100.0 (81.5 −100.0)	100	87.5	88.0
214	1.27	0.690 ± 0.170 (0.476−0.858)	57.1 (18.4 – 90.1)	100.0 (81.5 −100.0)	100	87.5	88.0
223	1.27	0.690 ± 0.170 (0.476−0.858)	57.1 (18.4 – 90.1)	100.0 (81.5 −100.0)	100	87.5	88.0
245	1.27	0.690 ± 0.170 (0.476−0.858)	57.1 (18.4 – 90.1)	100.0 (81.5 −100.0)	100	87.5	88.0

ROC, operating characteristic curve; AUC, area under curve. ADC, apparent diffusion coefficient; SE, standard error; CI, confidence interval; ROI, region of interest; PPV, positive predictive value; NPV, negative predictive value; ACC, accuracy.

## DISCUSSION

DWI has been widely used to detect and evaluate various tumors by noninvasively measuring ADC [16]. In pancreas imaging, a large number of studies have demonstrated significantly lower ADCs in PDAC than those in benign pancreas tissue [17–26]. As a supplement technique to conventional MR imaging, DWI improved the sensitivity of PDAC detection (with sensitivity and specificity up to 97% and 92%, respectively) [27]. However, quantitative ADC failed to differentiate the solid pancreatic lesions, due to the fact that a wide overlap in mean ADC values for different types [8, 28]. Takeuchi M, *et al.* [9] and Lee SS, *et al.* [8] reported that lower ADC values were observed in MFCP compared to PDAC. Contrarily, another two studies found the mean ADC values of PDAC was significantly lower than those of MFCP [4, 10]. In our study, no significant difference was observed between the mean ADC values of MFCP and PDAC, which was similar to the reported results of Wiggermann P, *et al.* [11] and Sandrasegaran K, *et al.* [12]. Areas of fibrosis and focal inflammatory reactions might explain the difficulty in differentiating MFCP from PDACs by using the mean ADCs [11].

In the ADC measurements for pancreatic lesions, although no formal recommendation was reported before, a minimum size of 100 mm^2^ was commonly used [7]. Our results showed that ROI size has remarkable influence on the differentiation between NP, PDAC and MFCP, confirmed by the fact that the mean ADCs of PDAC were significantly lower than those of NP (all *P* < 0.05) when with ROI ≥ 55 mm^2^, and the mean ADCs of MFCP were significantly lower than those of NP (all *P* < 0.05) when ROI ≥ 99 mm^2^. Additionally, according to the ROC analyses results, a ROI size of ≥ 214 mm^2^ is recommended for the differentiation of PDAC and NP; and a ROI size of ≥ 99 mm^2^ is recommended to differentiate between MFCP and NP.

Three ROI methods, including the whole-volume ROI, single-slice ROI and small solid-sample ROI approaches, have been used to obtain ADC measurements from tumors [8–15, 17–28]. In our previous studies, we found that despite its large inter-observer variability, small solid-sample ROIs on tumors provided greater diagnostic performance in the assessment of PDAC compared with single-slice and whole-volume ROI methods [29]. Additionally, it is difficult to perform ADC measurements with whole-volume or single-slice ROI method in many tumors of patients with PDAC because of the unclear boundaries of tumors on DWI images [23, 29, 30]. Solid-sample ROI is the most commonly used approach for ADC measurements of PDAC [8, 10, 11, 18, 20, 22, 23, 25]. The ROIs in the lesions are delineated to avoid pancreatic ducts, cystic lesions, and imaging artifacts. Therefore, the small solid-sample ROI approach was used in this study.

Our study has several limitations. Firstly, the number of patients and control group was small, and only 7 patients were included in MFCP group. A larger sample size is required to confirm our results in future work. Secondly, in order to decrease the imaging variables and keep them as constant and homogeneous as possible, all the MR examinations of all patients were performed with the same imaging protocols and parameters on a 3.0-T MRI system from a single vendor, whereas such an ideal scenario may not be available in actual daily clinical practice. In the future, well-designed, multicenter studies are needed to better determine the most appropriate usage of ADC in the field of pancreatic disease. Thirdly, our DWI experiments were performed with a relatively low b value (600 s/mm^2^) to minimize motion artifacts and to improve the signal-to-noise ratio in pancreas, while it has been shown that the use of higher b values may be more sensitive to reflect true diffusion [19]. Furthermore, only two b values (0 and 600 s/mm^2^) were performed in our study to reduce the scan time in the clinical setting, although ideally multiple b values should be used for more accurate measurements of ADC values [31, 32].

In conclusion, this focused DWI study demonstrated that ROI size had a considerable influence on the differentiation between NP, PDAC and MFCP at 3.0T. A ROI size of ≥ 214 mm^2^ is recommended for the differentiation between PDAC and NP; and a ROI size of ≥ 99 mm^2^ is recommended to differentiate between MFCP and NP.

## MATERIALS AND METHODS

### Study design and population

The study was composed of phantom experiments and *in vivo* scans of healthy subjects and patients. A round water phantom (18-cm in diameter, 20^°^C) was imaged to validate the reliability of our scanner and demonstrate the results of the ideal mean ADC values with different ROIs as a reference measurement. All experiments were performed on a 3.0-T MRI system (Signa HDxt, GE Healthcare, Milwaukee, WI, USA) with a 40 mT/m maximum gradient strength and a peak slew rate of 150 T/m/s. A body coil was used for signal transmission and an eight-element phased array coil placed over the abdomen was used for signal acquisition.

This retrospective study was reviewed and approved by the ethics committee of our hospital, and informed consent was waived from all the participants. Between January 2014 and February 2017, sixty-four patients with pathology-proven PDAC, seven patients with pathology-proven MFCP and eighteen healthy volunteers were included in the study. Mean age of the healthy volunteer group was 46.8 ± 12.0 years (range: 27–65 years), whereas mean age of the PDAC and MFCP groups was 61.1 ± 8.7 years; years (range: 40–78 years) and 47.3 ± 10.7 years (range: 32–66 years), respectively. The mean lesion size was 37 ± 9 mm (range, 21–70 mm) and 39 ± 12 mm (range, 27–64 mm) for PDAC and MFCP, respectively.

### *In Vivo* imaging

All of the 89 participants were preoperatively examined with conventional MRI protocols and transversal respiratory triggered single-shot echo-planar DWI (diffusion gradients along the physical x, y, and z axes), using b values of 0 and 600 s/mm^2^. Spectral selective presaturation with inversion recovery was used to achieve fat saturation. The main scan parameters and the scanning order of sequences were presented in Table 4. Out of the whole cohort, only 71 patients underwent contrast-enhanced liver acceleration volume acquisition (LAVA), which was performed with Gadopentetate Dimeglumine injection (physiological saline, 10 – 15 ml; media, 0.2 – 0.3 ml/kg) at the end of the study.

**Table 4:** The main parameters of MRI protocol

Protocols	TR/TE(ms)	FOV(mm)	Matrix	Thickness/gap(mm)	Flip angle(°)	Slices	NEX	Bandwidth(kHz)	Speed factor
MRCP	7000/1253.4	300 × 300	288 × 288	64/0	-	6	0.92	31.2	-
T2WI	6316/73.8	360 ~ 400	320 × 192	5/1	90	20	2	83.3	2
DWI	6000/58.6	380 × 304	128 × 96	5/1	90	20	2/4^*^	250	2
T1WI	2.5/1.1	440 × 418	256 × 180	2.5/0	11	84	0.70	125	2

^*^NEX = 2 for DWI at b_0_, NEX = 4 for DWI at b_600_.

MRCP, magnetic resonance cholangiopancreatography; T2WI, T2-weighted imaging; DWI, diffusing weighted imaging; T1WI, T1-weighted imaging.

### Phantom experiments

In the phantom study, the phantom was scanned using single-shot echo-planar DWI. The scan parameters were TR = 3000 ms, TE = 58.3 ms, FOV = 38.0 × 30.4 cm^2^, matrix = 128 × 96, slice/gap thickness = 5 mm / 1.5 mm and NEX = 1 and 4 for b_0_ and b_600_, respectively.

### Data analysis

**Figure 3:**
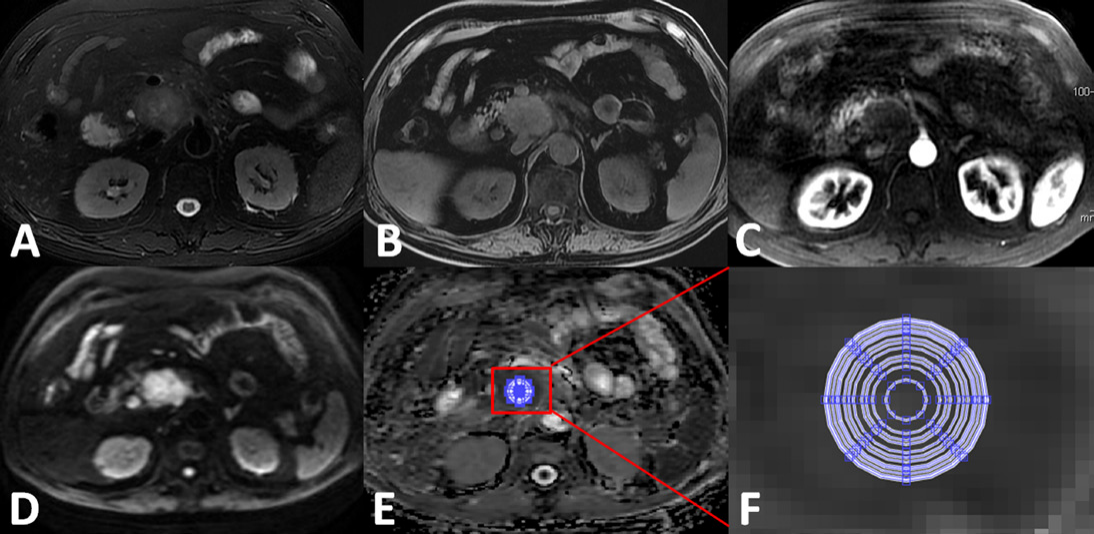
Apparent diffusion coefficient (ADC) measurements for pancreatic ductal adenocarcinoma at the head of the pancreas (**A**) Axial T2WI; (**B**) Axial precontrast T1WI; (**C**) Axial contrast-enhanced arterial phase T1WI demonstrating the hypovascularity of the mass; (**D**) DWI image (b = 600 s/mm^2^) clearly demarcated hyperintensity while compared with the surrounding pancreas tissues; (**E**) ADC map; (**F**) zoomed-in ADC map that indicates 12 concentric round ROIs (areas 15, 46, 55, 82, 99, 121, 134, 161, 189, 214, 223, and 245 mm^2^ were used for mean ADC measurements.

**Figure 4:**
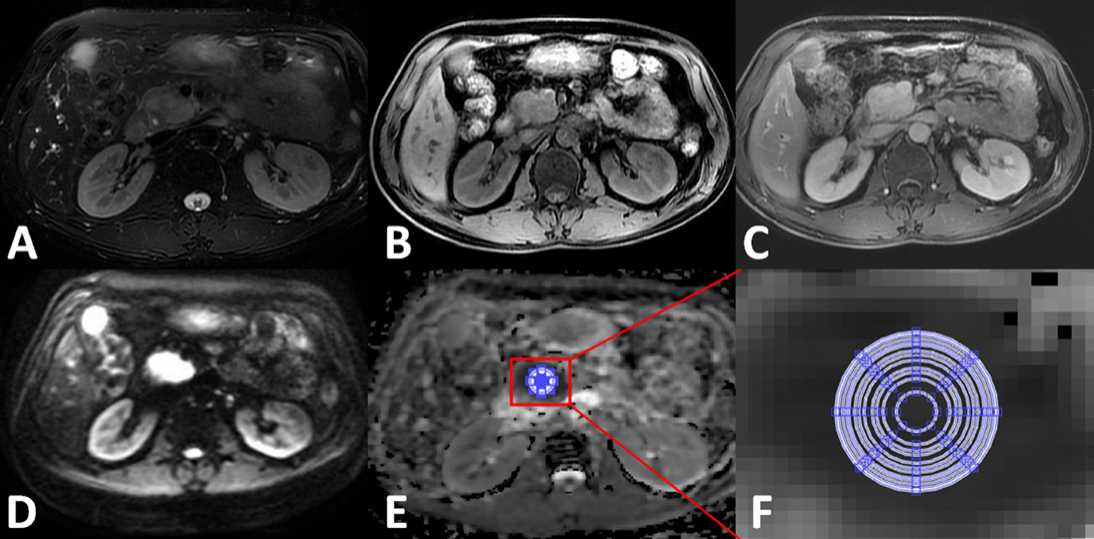
Apparent diffusion coefficient (ADC) measurements for mass-formed chronic pancreatitis at the head of the pancreas (**A**) Axial T2WI; (**B**) Axial precontrast T1WI; (**C**) Axial contrast-enhanced arterial phase T1WI; (**D**) DWI image (b = 600 s/mm^2^) clearly demarcated hyperintensity while compared with the surrounding pancreas tissues; (**E**) ADC map; (**F**) zoomed-in ADC map that indicates 12 concentric round ROIs (areas 15, 46, 55, 82, 99, 121, 134, 161, 189, 214, 223, and 245 mm^2^ were used for mean ADC measurements.

ADC maps were derived from the DWI images using a monoexponential model (ADC = [ln(SI_b0_/SI_b600_)]/600) on a workstation (Function V9.4.05, Advanced Workstation 4.4, GE Healthcare). The anonymous MR images of each participant were sorted in a random order. A homemade software was used to measure the mean ADC value within each of 12 concentric round ROIs (areas: 15, 46, 55, 82, 99, 121, 134, 152, 161, 189, 214, 223, and 245 mm^2^ with pixel numbers: 7, 21, 25, 37, 45, 61, 69, 73, 86, 97, 101 and 111, respectively) drawn on the solid part of the mass of lesions and the head of NP. ADC values were measured by two observers (with 11 and 6 years of experience in abdominal radiology) (Figures 3 and 4), avoiding pancreatic ducts and cystic lesions by referring to other MRI images such as T2WI or T1WI. Water phantom was used to calculate the ideal mean ADCs with 10 times random measurements as a reference.

### Statistical analysis

Statistical analyses were performed using Medcalc software (Version 13.0.0.0, MedCalc software). The mean ADCs obtained from the 12 different-sized ROIs were compared by one-way repeated analysis of variance (ANOVA) for each group of PDAC, MRCP or NP. *P* < 0.05 was considered as statistically significant. The comparison of mean ADC values for each ROI size among the three groups were analyzed using Kruskal-Wallis tests and a test for pairwise comparison of subgroups were conducted according to Conover. In addition, receiver operating characteristics (ROC) analyses were used to identify the diagnostic performances of the mean ADCs for the differentiation between NP, PDAC and MFCP.
